# LC-AMP-I1, a novel venom-derived antimicrobial peptide from the wolf spider *Lycosa coelestis*

**DOI:** 10.1128/aac.00424-24

**Published:** 2024-12-02

**Authors:** Junyao Wang, Xi Liu, Yuxin Song, Zhonghua Liu, Xing Tang, Huaxin Tan

**Affiliations:** 1 Institute of Biochemistry and Molecular Biology, Hengyang Medical College, University of South China 34706 , Hengyang, China; 2 The National and Local Joint Engineering Laboratory of Animal Peptide Drug Development, College of Life Sciences, Hunan Normal University 655977 , Changsha, China; 3 Hunan Key Laboratory for Conservation and Utilization of Biological Resources in the Nanyue Mountainous Region, College of Life Sciences, Hengyang Normal University 12573 , Hengyang, China; University of Pittsburgh School of Medicine, Pittsburgh, Pennsylvania, USA

**Keywords:** antimicrobial peptide, *Lycosa coelestis*, antibacterial activity, drug resistance, biofilm, conventional antibiotic

## Abstract

Antibiotic resistance has become a critical concern in recent years, and antimicrobial peptides may function as innovative antibacterial agents to address this issue. In this work, we identified a novel antimicrobial peptide, LC-AMP-I1, derived from the venom of *Lycosa coelestis*, demonstrating substantial antibacterial properties and minimal hemolytic activity. LC-AMP-I1 was subjected to additional assessment for antibacterial efficacy, anti-biofilm properties, drug resistance, stability, and cytotoxicity *in vitro*. It exhibited comparable antibacterial efficacy to melittin against six common clinical multidrug-resistant bacteria, effectively inhibiting biofilm formation and disrupting established biofilms. Additionally, LC-AMP-I1 demonstrated minimal bacterial resistance, excellent stability, negligible mammalian cell toxicity, low hemolytic activity, and appropriate selectivity for both normal and tumor cells. When combined with traditional antibiotics, LC-AMP-I1 exhibited additive or synergistic therapeutic effects. In a neutropenic mouse thigh infection model, LC-AMP-I1 exhibited a therapeutic effect in inhibiting bacterial proliferation *in vivo*. The mechanistic investigation indicated that LC-AMP-I1 could influence bacterial cell membrane permeability at low concentrations and directly disrupt structure-function at high concentrations. The results of this work indicate that LC-AMP-I1 may function as a viable alternative to traditional antibiotics in addressing multidrug-resistant bacteria.

## INTRODUCTION

The discovery and application of antibiotics marked the beginning of an era in which previously fatal infectious diseases could be treated (1). Nevertheless, the efficacy of antibiotics is progressively diminishing, and resistance is rapidly developing among microorganisms, as a result of the frequent and inappropriate administration of these drugs to both humans and animals (2, 3). Currently, antimicrobial resistance is recognized by the World Health Organization as one of three major threats to public health (4). The search for alternative antibiotics is thus imperative.

Natural antimicrobial peptides (AMPs) are essential components of the innate immune system of all living organisms, safeguarding the body against various pathogens (5). Those peptides are generally defined by their small size, simple structure, excellent biocompatibility, and minimal drug resistance. They demonstrate efficacy against cancer cells, bacteria, viruses, and parasites, in addition to inhibiting biofilm formation (6). As of now, over 3,900 natural and synthetic antimicrobial peptides have been recorded in the antimicrobial peptide (APD3) database (https://aps.unmc.edu/home). Most antimicrobial peptides exhibit positive charge and amphiphilic characteristics, primarily inducing bacterial death through interactions with cell membranes (7, 8). This differs from the inhibitory mechanism of traditional antibiotics, which exert destructive effects on bacterial metabolic processes (9, 10). Consequently, antimicrobial peptides represent promising candidates for the creation of new antibiotics (11, 12).

Spiders, with more than 50,000 species, are recognized as diverse predators in the phylum Arthropoda (World Spider Catalogue, 24th edition). Spider venoms are composed of intricate mixtures that include a variety of biologically active peptides (13, 14). Some studies have reported on the venom composition of *Lycosidae* spiders, which includes neurotoxic peptides featuring multiple disulfide bridges and antimicrobial peptides lacking cysteine residues (15–18). In prior research, Lycosin-I was isolated from the venom of *Lycosa singoriensis*, demonstrating significant antibacterial properties (19) and potential applications in antitumor therapies and multifunctional anticancer nanomaterials (20, 21). Furthermore, 52 antimicrobial peptides from eight distinct families (families A–H) were identified in the venom of *Lycosa sinensis* (22). LS-AMP-E1 and LS-AMP-F1, derived from the venom of the wolf spider *L. sinensis*, demonstrated the capacity to inhibit the growth of clinically isolated multidrug-resistant strains and sterilize bacteria synergistically when used in conjunction with conventional antibiotics (22, 23).

Recently, 98 neurotoxin peptides were identified from the venom of the Chinese wolf spider *Lycosa coelestis* through transcriptomics. These peptides exhibited 2–14 cysteines, forming 1–7 disulfide bridges, and were categorized into 11 families (24). Only two AMPs have been identified in *L. coelestis* venom: XYP1, which exhibits anti-toxoplasma activity, and LC-AMP-F1, known for its antibacterial and antibiofilm properties (25, 26). In order to identify additional venom-derived AMPs with potent and low toxicity from *L. coelestis*, we employed a combined approach of peptidomics and transcriptomics of *L. coelestis* venom to isolate linear cationic AMPs devoid of disulfide bonds. In this work, we have successfully identified a novel AMP from *L. coelestis* venom, named LC-AMP-I1. This cationic peptide demonstrated significant stability, extensive antibacterial efficacy, and low cytotoxicity in both *in vitro* and *in vivo* studies. The mechanism and therapeutic potential of LC-AMP-I1 were further investigated. The findings of this study suggest that LC-AMP-I1 may serve as a promising alternative to conventional antibiotics.

## RESULTS

### Purification and characterization of LC-AMP-I1

**Fig 1:**
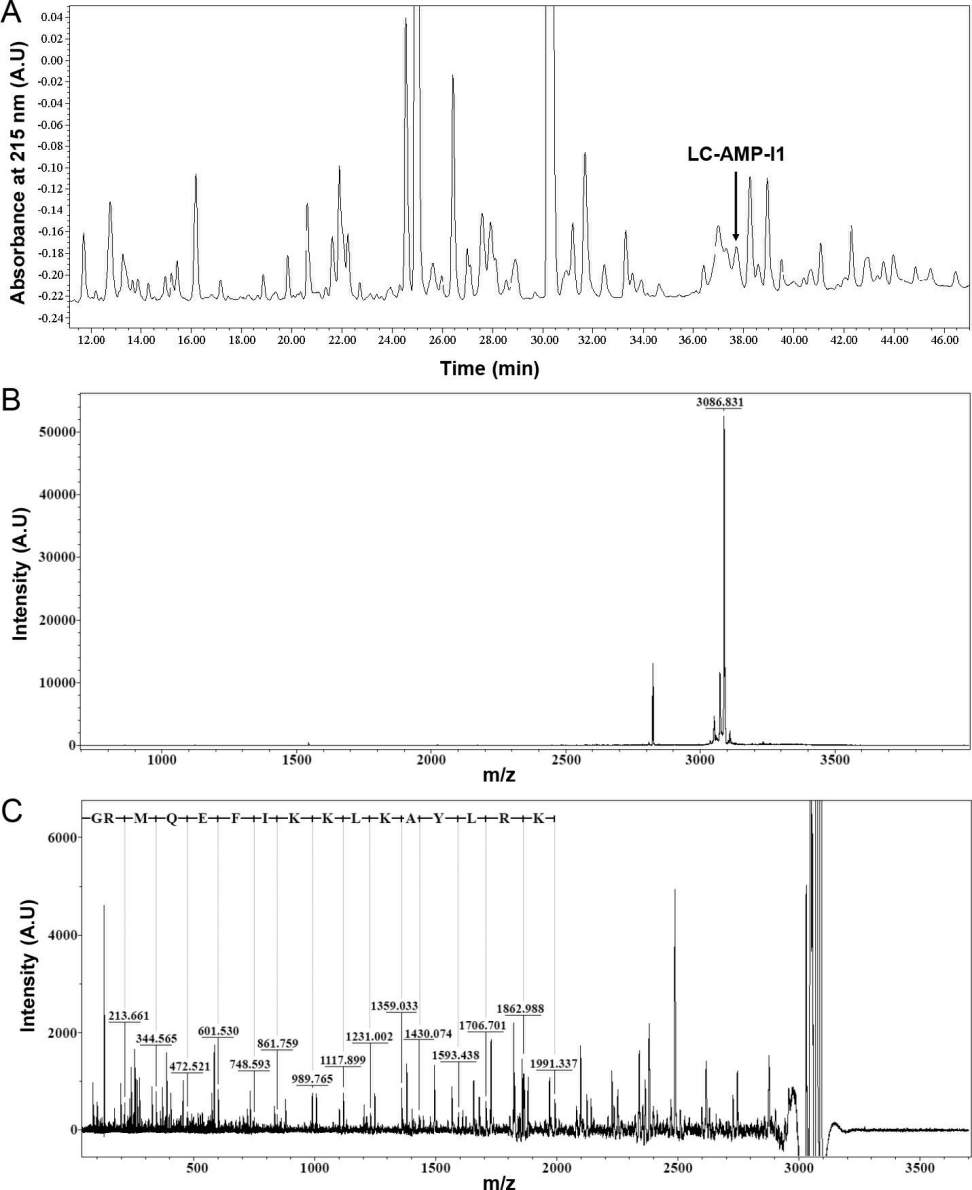
Isolation and purification of LC-AMP-I1. (**A**) RP-HPLC profile of peptides from *L. coelestis* venom. An enlarged view of Fig. S1. The elution time of the fraction containing LC-AMP-I1 marked by the arrow in panel A is 37.73 min. (**B**) MS spectrum in the reflector mode of the fraction (including LC-AMP-I1 with parent ion *m/z* 3,086.831) eluted at retention time (37.73 min) on RP-HPLC. (**C**) MS/MS spectrum of LC-AMP-I1 with parent ion *m/z* 3,086.831 from panel B. The amino-terminal amino acid sequences with GRMQEFIKKLKAYLRK were derived from b-series ions.

As in our earlier research, the alignment of transcriptomics and proteomics data together was used to determine the LC-AMP-I1 sequence (22, 23). In short, we started by building a cDNA library of the venom gland of *L. coelestis*. The AMP sequences in the library were categorized into nine distinct antimicrobial peptide families (families A–I) based on the sequence characteristics and homology of cationic antimicrobial peptides. On the other hand, we separated the peptide components in the spider venom using reversed-phase high-performance liquid chromatography (RP-HPLC) and identified each fraction using matrix-assisted laser desorption ionization-time of flight mass spectrometry (MALDI-TOF MS). A top-down strategy MALDI-TOF MS/MS sequencing was implemented for the single components that were obtained. Through the analysis and comparison of sequence data derived from both methodologies, we identified a novel cationic antimicrobial peptide, designated as LC-AMP-I1. This peptide was found at the elution peak of 37.73 min (Fig. 1B) with a monoisotopic mass (M + H)^+^ of 3,086.83 Da. The amino-terminal sequence of LC-AMP-I1, GRMQEFIKKLKAYLRK, was obtained from b-series ions (Fig. 1C). This sequence was derived from the LC-AMP precursor 3 and was categorized within the LC-AMP I family in the transcriptome data. This precursor can be cleaved to yield four mature peptides during maturation: LC-AMP-I1, LC-AMP-D1, LC-AMP-D2, and LC-AMP-D3. The mature peptide of LC-AMP-I1 comprises 25 amino acids (GRMQEFIKKLKAYLRKMKEKFSQIS-NH_2_) with an amidated carboxyl terminus. The calculated molecular mass (3,086.72 Da) corresponds with the observed mass (Fig. 1B).

**Fig 2:**
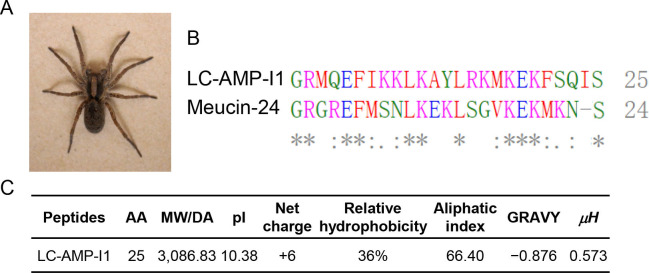
Characterization of LC-AMP-I1. (**A**) Adult *L. coelestis* spider. (**B**) Multiple sequence alignment of LC-AMP-I1 with homology AMPs. “*” indicates the amino acid residues at this position are identical. “:” indicates the amino acid residues at this position are strongly similar. “.” indicates the amino acid residues at this position are weakly similar. No marking means that the amino acid residues at this position are completely different. (**C**) Amino acid sequences and physical characteristics for LC-AMP-I1.

Sequence homology analysis was performed using the APD database with the collection of known antimicrobial peptides, revealing that the peptide Meucin-24 (from *Mesobuthus eupeus*) exhibits the highest sequence similarity of 44% to LC-AMP-I1.(Fig. 2B) Further sequence analysis and predictions were conducted in ProtParam. The pI value, aliphatic index, and grand average of hydropathicity of LC-AMP-I1 were determined to be 10.38, 66.40, and −0.876, respectively.(Fig. 2C)

### Secondary structure of LC-AMP-I1

**Fig 3:**
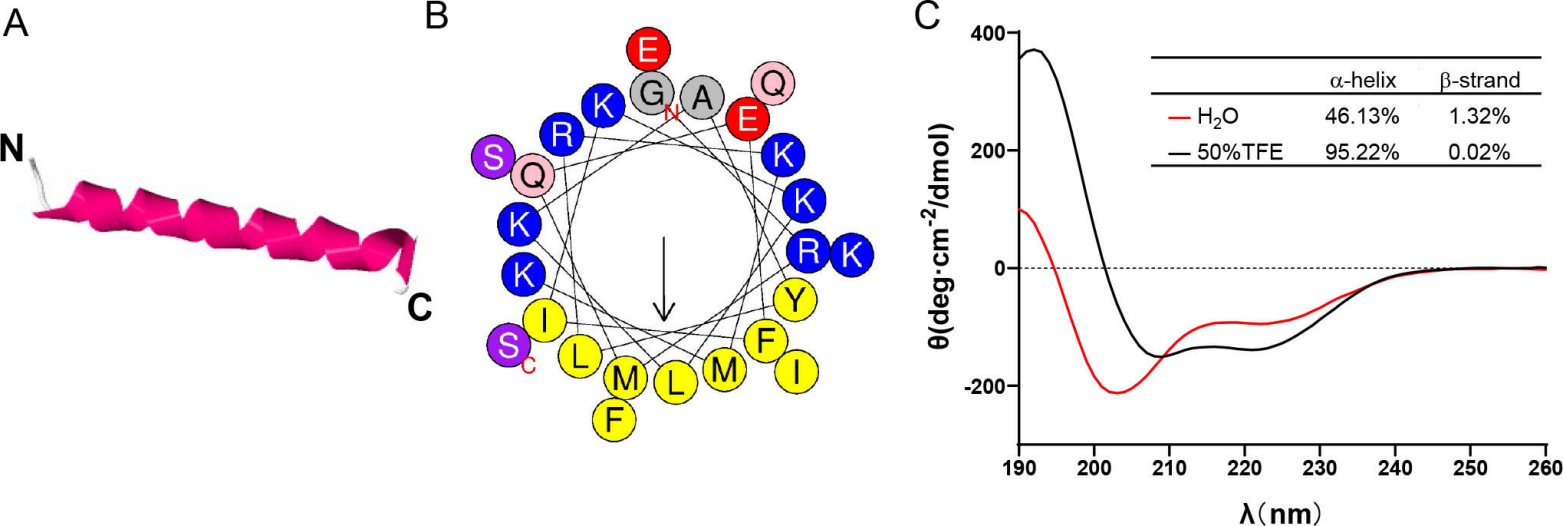
Structural analysis of LC-AMP-I1. (**A**) Helical diagrams for LC-AMP-I1. The hydrophobic residues are presented in yellow color, positively charged hydrophilic residues in blue, the noncharged polar residue in purple, and negatively charged hydrophilic residue in red. (**B**) Three-dimensional structures of LC-AMP-I1. (**C**) Circular dichroism spectra of LC-AMP-I1. The secondary structure data of LC-AMP-I1 were analyzed by the K2D3 online analysis program.

The amphiphilicity of antimicrobial peptides has been recognized as a crucial determinant of their antimicrobial activity (27). Hence, to evaluate the amphiphilicity of the peptide by predicting its amino acid residue distribution, we utilized HeliQuest to generate a helical wheel of LC-AMP-I1. A standard amphiphilic helical conformation and stability can be achieved by organizing amino acid residues of varying polarities in the LC-AMP-I1 sequence, as illustrated in Fig. 3A. Furthermore, the three-dimensional helical configuration of LC-AMP-I1 was predicted using the protein structure modeling software I-TASSER, as illustrated in Fig. 3B. To confirm this prediction, we examined the secondary structure of LC-AMP-I1 in various solvents using circular dichroism (CD) spectroscopy. The CD spectra of LC-AMP-I1 in water exhibited a typical randomly coiled peak shape, as depicted in Fig. 3C (with a negative peak at 200 nm) (28). In TFE/H_2_O, which simulates a cell membrane environment, a typical α-helical conformation was formed, as indicated by a positive absorption peak near 190 nm and negative absorption peaks at 208 and 222 nm. In detail, the estimation of these secondary structures of the peptide using the K2D3 software revealed that the α-helical content of LC-AMP-I1 was 46.13% and 95.22% in H_2_O and TFE/H_2_O, respectively. The above results indicated that LC-AMP-I1 exhibited an amphiphilic α-helical structure, similar to classic cationic antimicrobial peptides, which strongly suggested that LC-AMP-I1 possessed potent antimicrobial activity.

### Antibacterial activities of LC-AMP-I1

**Fig 4:**
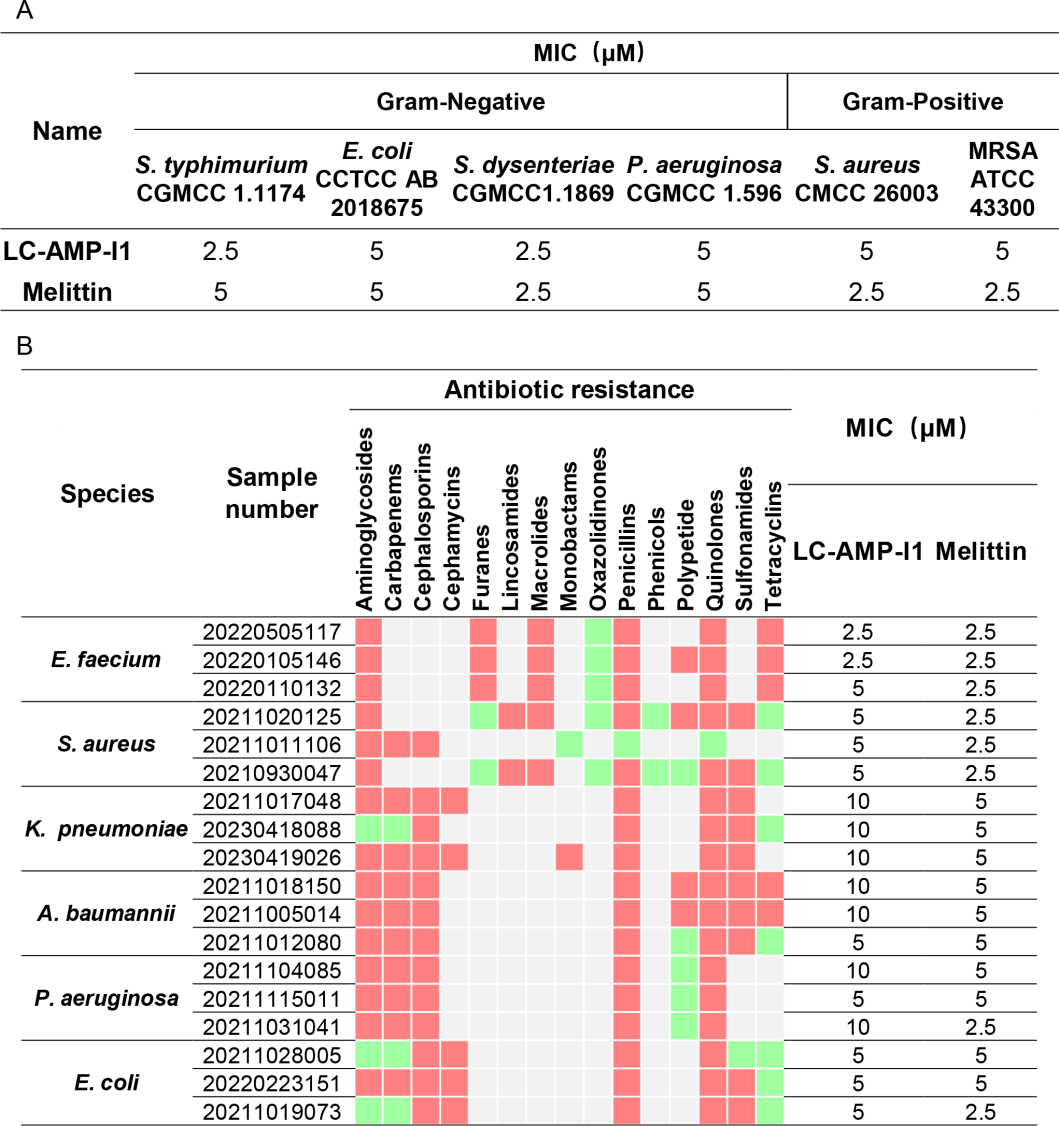
The MIC of LC-AMP-I1 against standard strains (**A**) and clinical bacterial strains (**B**). Bacteria were susceptible to all (green boxes) or intermediate/resistant to at least one (red boxes) of the antibiotics per class. Gray boxes are shown if the susceptibility to agents in that class is not assessed. MIC was defined as the minimum concentration to kill the bacterial strains completely.

The antimicrobial activity of LC-AMP-I1 was assessed against a variety of reference microorganisms and clinical isolates. As a positive control, melittin, a membrane-breaking antimicrobial peptide derived from honeybee venom, was prepared. The minimum inhibitory concentrations (MICs) of melittin and LC-AMP-I1 against standard strains, two Gram-positive and four Gram-negative, were displayed in Fig. 4A. LC-AMP-I1 and melittin exhibited similar levels of antimicrobial efficacy, as evidenced by their MICs ranging between 2.5 and 5 μM. Notably, the MIC of LC-AMP-I1 against methicillin-resistant *Staphylococcus aureus* (MRSA) was 5 μM. To conduct a comprehensive assessment of the peptide’s efficacy against antibiotic-resistant bacteria, we examined its activity against 18 clinically isolated strains of multidrug-resistant bacteria from the ESKAPE panel (clinical details are provided in Table S1), a group of bacteria known for their potential resistance to various antibiotics and their association with significant nosocomial infections (29). As shown in Fig. 4B, the MICs of LC-AMP-I1 against these drug-resistant bacteria were between 2.5 and 10 μM. It is noteworthy that these isolates exhibited resistance to at least four commonly used antibiotics. This finding supported the hypothesis that, like melittin, LC-AMP-I1 would have peculiar mechanisms and inhibitory activities in contrast to traditional antibiotics.

**Fig 5:**
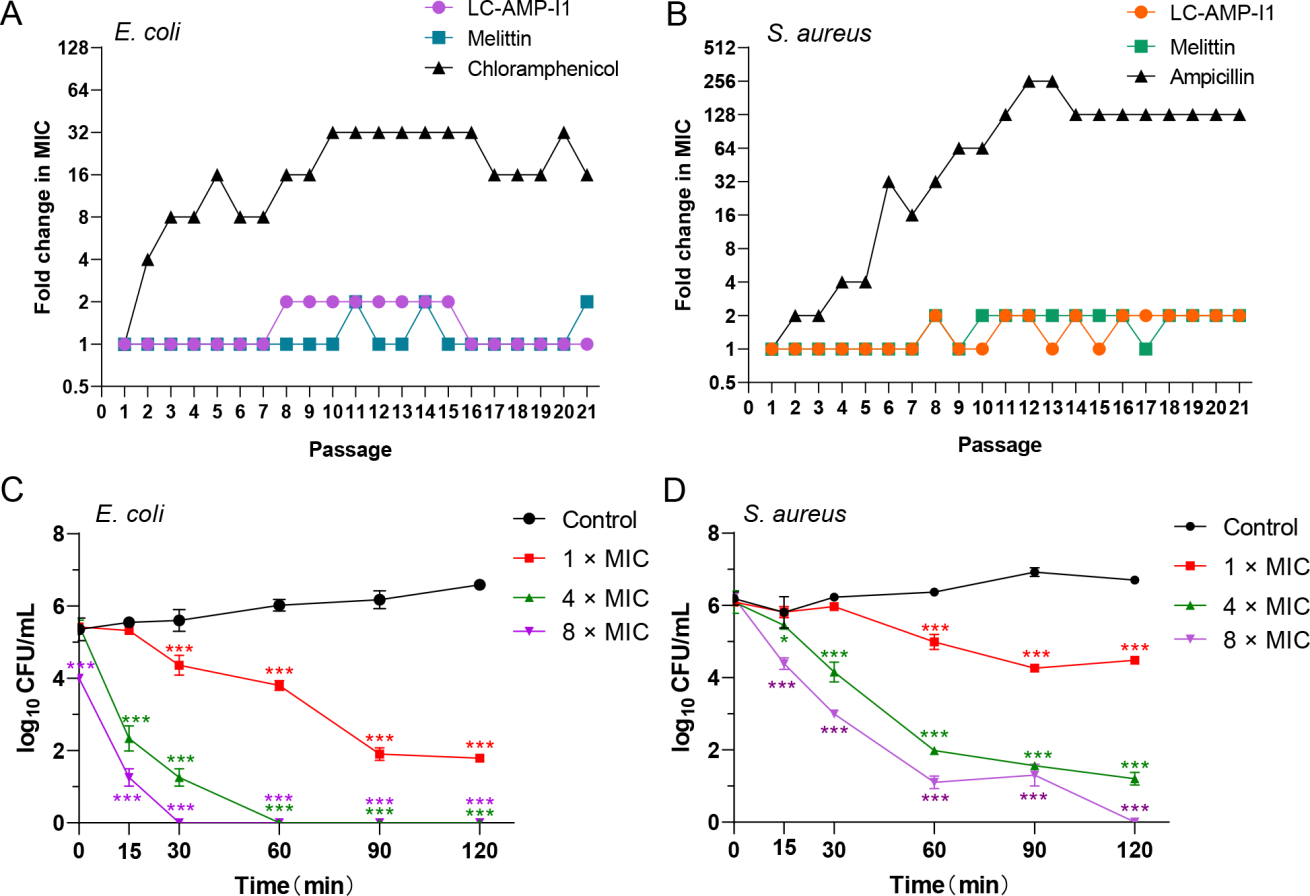
Resistance induction and killing curves of LC-AMP-I1. The MIC value of LC-AMP-I1 against *E. coli* CCTCC AB 2018675 (**A**) and *S. aureus* CMCC 26003 (**B**) after 21 consecutive days of treatment. Bacterial cells were exposed to peptides at sublethal concentrations at 37°C for 18 hours to determine the MIC of the peptides. The MIC of each passage of peptide was recorded for 21 consecutive days. Time-killing kinetics of LC-AMP-I1 against *E. coli* CCTCC AB 2018675 (**C**) and *S. aureus* CMCC 26003 (**D**). Controls were bacterial cells treated with phosphate-buffered saline. MIC = 5 μΜ. The experiment was conducted in triplicate. *P < 0.05 and ***P < 0.001.

On the other hand, we assessed the potential resistance to LC-AMP-I1 that could be developed in two bacterial strains (*Escherichia coli* and *S. aureus*). The MICs were determined after treating with a 1/2 MIC of peptides or antibiotics for 21 days in a row. As illustrated in Fig. 5A, during 21 consecutive passages, the MICs of LC-AMP-I1 and melittin against *E. coli* were consistently stable. In contrast, exposure to chloramphenicol resulted in a notable increase in resistance in *E. coli* after three successive passages. The MIC of chloramphenicol escalated to 32 times the initial effective dose after 10 passages. In the case of *S. aureus*, this difference was more noticeable. As presented in Fig. 5B, *S. aureus* maintained a high level of susceptibility to LC-AMP-I1 and Melittin across 21 successive drug incubations and cultures. However, the MIC of ampicillin exhibited a significant rise of 256-fold after 13 days of treatment.

In addition to the ability to effectively suppress bacterial growth at low doses, LC-AMP-I1 could quickly and efficiently kill bacteria at high concentrations. As proved in Fig. 5C, *E. coli* could be entirely eradicated after being treated with LC-AMP-I for 60 min at a peptide concentration of fourfold MIC (Fig. 5C). This rapid bactericidal effect demonstrated a distinct dependence on concentration. As to *S. aureus*, even though the time–kill curve of LC-AMP-I1 was slowing down, the peptide concentration of fourfold MIC was still able to lower the bacterial concentration by about four orders of magnitude at 60 min (Fig. 5D).

### Biofilm inhibition and eradication activities of LC-AMP-I1

**Fig 6:**
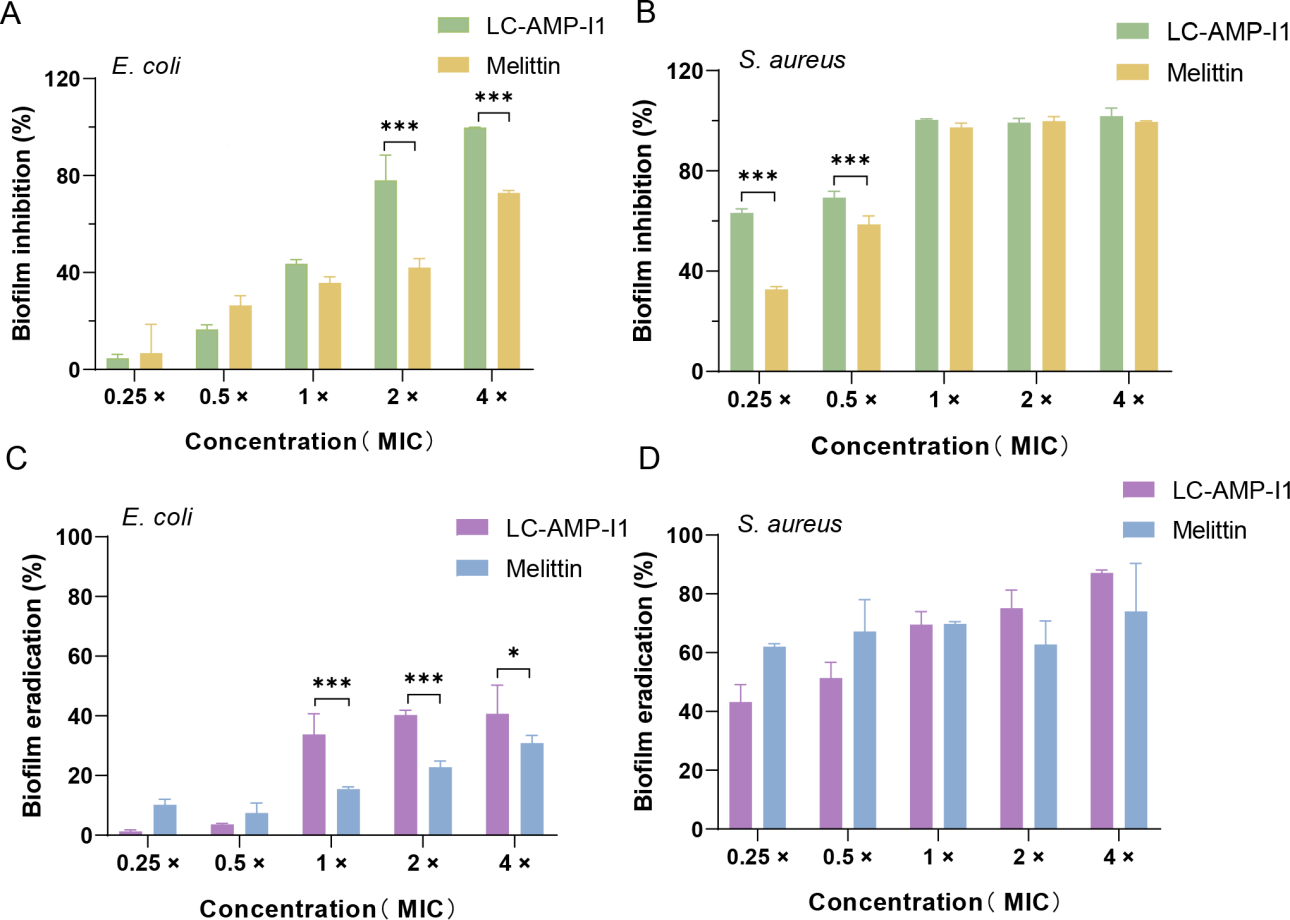
Biofilm inhibition and eradication activities of LC-AMP-I1. Inhibition activities of LC-AMP-I1 on *E. coli* CCTCC AB 2018675 (**A**) and *S. aureus* CMCC 26003 (**B**) biofilms. Eradication activities of LC-AMP-I1 on *E. coli* CCTCC AB 2018675 (**C**) and *S. aureus* CMCC 26003 (**D**) biofilms. Each measurement was performed in triplicate. Data are presented as mean values of 3 standard deviations. *P < 0.05 and ***P < 0.001.

Investigations into the management of drug-resistant bacteria focus on the inhibition and eradication of biofilms, which are the principal contributors to chronic and recurrent infections as well as bacterial drug resistance (30, 31). In this work, we found that LC-AMP-I1 could reduce and eliminate biofilm formation of *E. coli* and *S. aureus* in a dose-dependent manner. Figure 6A shows that LC-AMP-I1 reduced biofilm formation in *E. coli* by 43.67% and melittin reduced biofilm formation by 35.79% at 1× MIC conditions (5 μΜ). The LC-AMP-I1 concentration necessary to completely block the *E. coli* biofilm was 4× MIC (20 μΜ), while melittin exhibited a 72.81% inhibition at this dosage. LC-AMP-I1 prevented 63.2% of biofilm formation in *S. aureus* at 0.25× MIC, whereas melittin inhibited 32.74% of biofilm formation. At a concentration of 1× MIC, the two peptides prevented biofilm formation by 100% and 97.4%, respectively (Fig. 6B).

The clearance of 24-hour mature biofilms by peptides is illustrated in Fig. 6C and D. The eradication of 1-day mature *E. coli* biofilms by LC-AMP-I1 and melittin was minimal. At a concentration of 1× MIC, LC-AMP-I1 and melittin eliminated only 33.83% and 15.49% of the *E. coli*-attached biofilm biomass, respectively. However, increasing the peptide concentration to 4× MIC resulted in a reduction of 40.74% and 30.87% in *E. coli* biofilm, respectively. At sub-MIC concentrations, LC-AMP-I1 and melittin eradicated 51.40% and 67.21% of *S. aureus* biofilms, respectively. LC-AMP-I1 removed 87.16% of the *S. aureus* biofilm at a concentration of 4× MIC biofilm biomass, respectively. However, increasing the peptide concentration to 4× MIC, melittin only removed 74.08%.

### Salt, temperature, and pH stability

**Fig 7:**
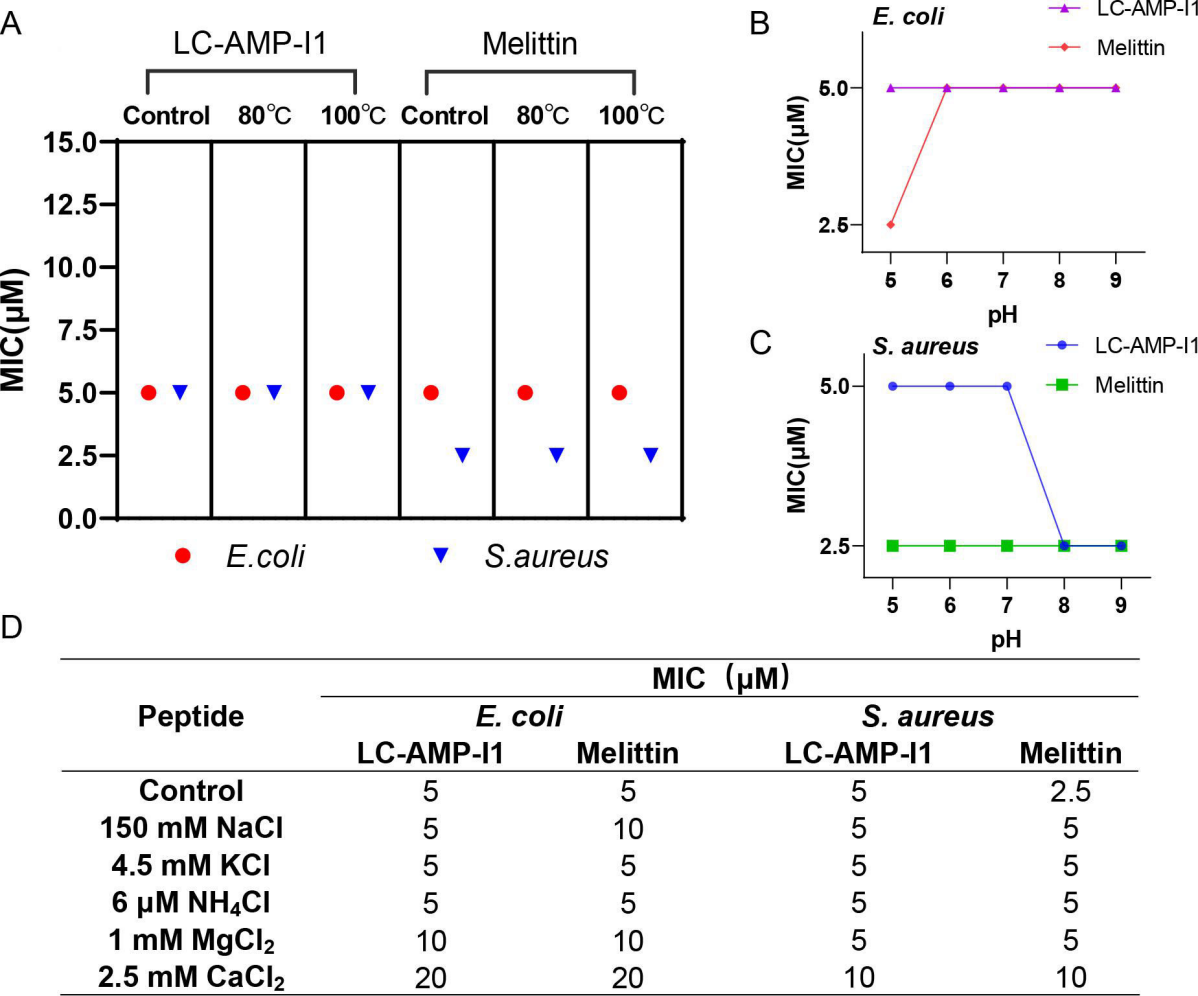
Stability of LC-AMP-I1 to high temperatures and influence of pH on the LC-AMP-I1 MIC. (**A**) Peptides were treated at 80°C or 100°C for 1 hour and then tested for antibacterial activity against *E. coli* CCTCC AB 2018675 and *S. aureus* CMCC 26003, respectively. The MIC of peptides against *E. coli* AB 2018675 (**B**) and *S. aureus* CMCC 26003 (**C**) in broth at pH 5–9. (**D**) Effects of salt ions on the antibacterial activity of the LC-AMP-I1 against *E. coli* CCTCC AB 2018675 and *S. aureus* CMCC 26003. The data were derived from three independent experiments (*n* = 3).

In general, the activities of proteins are less stable than those of inorganic small molecules because they are more sensitive to their surroundings. However, our study revealed that LC-AMP-I1’s antimicrobial effectiveness stayed impressively constant. Figure 7A indicates that the MICs of the LC-AMP-I1 solution against both *E. coli* and *S. aureus* remained unchanged following exposure to a water bath at 80°C or 100°C for 1 hour. Figure 7B and C demonstrates that the activity of LC-AMP-I1 remained mostly stable irrespective of the pH environment. Notably, the MIC of LC-AMP-I1 for *S. aureus* was reduced by 50% when the solution pH was above 8. This could be attributed to the alkaline environment, which enhanced the sensitivity of *S. aureus* cells to LC-AMP-I1. Furthermore, our findings indicated that increasing the ionic strength of the three solutions (150 mM NaCl, 4.5 mM KCl, or 6 µM NH_4_Cl) had no significant impact on the antibacterial activity of LC-AMP-I1 (Fig. 7D). However, as the surrounding concentration of Mg^2+^ and Ca^2+^ ions rose, the MIC of LC-AMP-I1 (or melittin) against *E. coli* was elevated by two and four times, respectively. The inhibitory activity of melittin against *S. aureus* in a solution containing double-charged ions exhibited a two- to fourfold rise in MIC, but the MIC of LC-AMP-I1 remained unchanged in the presence of Mg ^2+^ ions and was only increased by two times in the presence of Ca^2+^ ions. The reduction in antibacterial activity may be ascribed to the excess of cations that competitively inhibit the binding of cationic peptides to negatively charged bacterial surfaces (32, 33). Despite the inevitable reduction in the activities of two AMPs under double-charged ionic conditions, LC-AMP-I1 exhibited greater salt stability than melittin. The findings suggested that LC-AMP-I1 consistently and durably inhibited bacterial growth, likely due to its simple yet stable α-helical secondary structure.

### Selective cytotoxicity of LC-AMP-I1 *in vitro*

**Fig 8:**
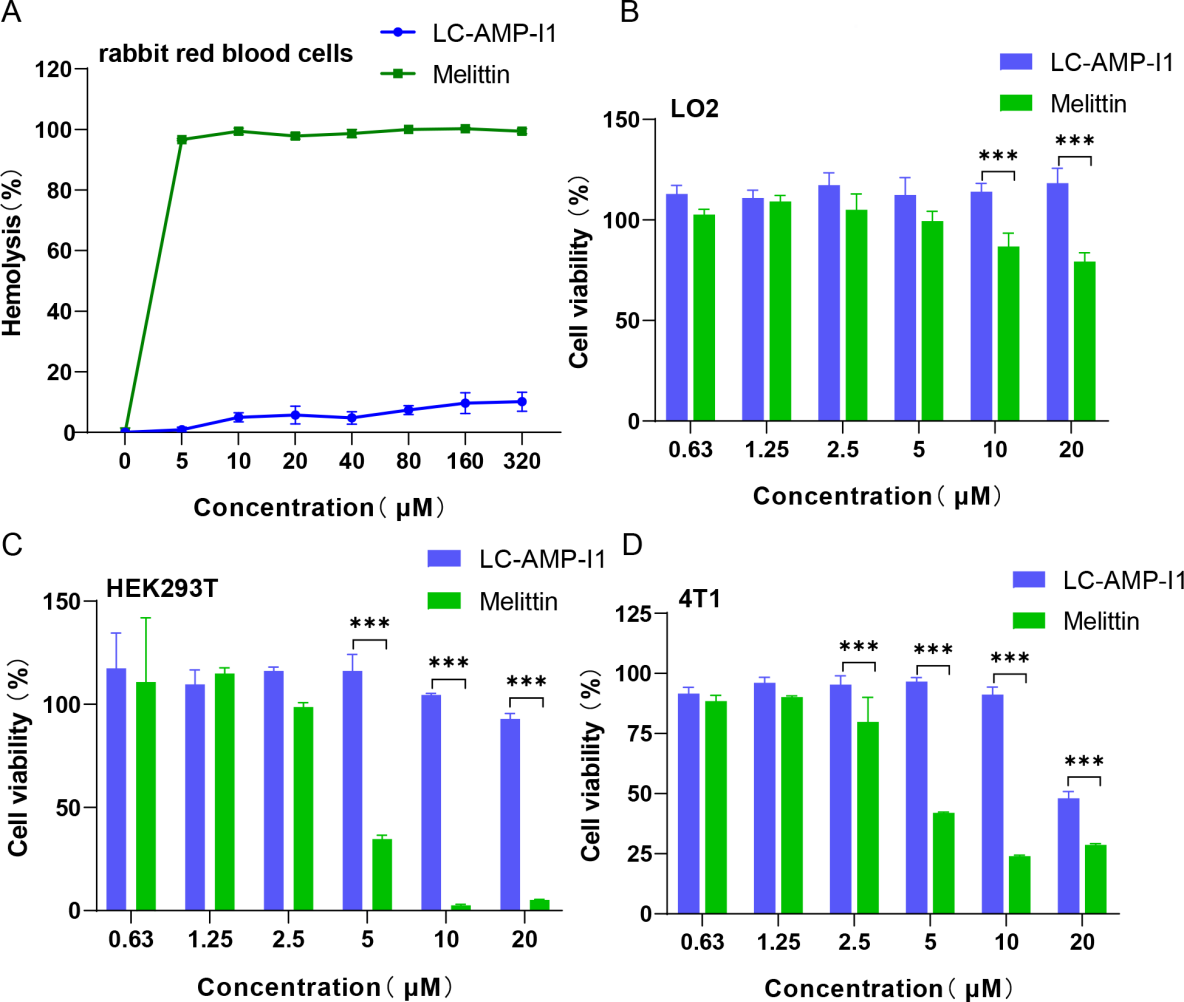
Hemolysis and cytotoxicity of LC-AMP-I1. (**A**) Hemolysis of LC-AMP-I1 against rabbit red blood cells. Cytotoxicity of LO2 cells (**B**), HEK293T cells (**C**), and 4T1 cells (**D**). The CCK-8 assay was used. Each measurement was performed in triplicate. Data are presented as mean values of three standard deviations. ***P < 0.001.

Four eukaryotic cells were selected to assess the biosafety of LC-AMP-I1, including three normal mammalian cells and a cancer cell. Unlike melittin, LC-AMP-I1 exhibited a remarkably reduced hemolytic activity. The hemolytic activity of LC-AMP-I1 was only 10.13% even at its maximum concentration of 320 μM (Fig. 8A). In contrast, at 5 μM, the hemolytic activity of melittin approached 100%. In addition, LC-AMP-I1 exhibited minimal toxicity to LO2 (Fig. 8B) and HEK293T (Fig. 8C) cells. It is noteworthy that LC-AMP-I1 exhibited cytotoxic activity against 4T1 cells (mouse breast cancer cells) (Fig. 8D) with an IC_50_ value of 19.58 μM (Fig. S3). Additionally, two tumor cell lines, HepG2 (human hepatocellular carcinoma cells) and HONE-1 (human nasopharyngeal carcinoma cells), were also tested in Fig. S4. LC-AMP-I1 showed low cytotoxicity toward the two tumor cells.

### Synergistic activity of LC-AMP-I1 with antibiotics

Combination therapy is often used to treat bacterial infections because the synergistic effects of antibiotics not only make drugs work better but also slow down the development of antibiotic resistance (34). The fractional inhibitory concentration index (FICI) was calculated to quantify peptide-conventional antibiotic synergy to assess LC-AMP-I1’s potential as a lead compound to antimicrobial drugs. The FICI values are presented in Table 1. The MICs of peptides and antibiotics were remeasured when combined at a series of fixed sub-MIC concentrations (Fig. S5). A statistically significant decrease in the MICs of each component for the tested bacteria was observed when LC-AMP-I1 was used in combination with both antibiotics. The combination of LC-AMP-I1 and levofloxacin showed notable synergistic inhibition against *S. aureus* (FICI ≤ 0.5) and a cumulative antibacterial effect against *E. coli*. Furthermore, both bacterial strains exhibited a significant decrease in antibiotic dosage due to the additive effect of LC-AMP-I1 and erythromycin (MIC of erythromycin decreased from 10 to 1.25 µg/mL for *E. coli* and from 0.08 to 0.04 µg/mL for *S. aureus*). The relatively low FICI values suggested that the synergistic therapeutic effect of LC-AMP-I1 combined with both antibiotics surpassed that of melittin.

**TABLE 1:** Synergistic activity of LC-AMP-I1 with antibiotics

Bacterial strain	Antibiotic	FIC in combination with:	
		LC-AMP-I1	Melittin
*E. coli*	Erythromycin	0.625	0.625
	Levofloxacin	0.75	1.0625
*S. aureus*	Erythromycin	0.625	1.125
	Levofloxacin	0.3125	0.3125

### Antimicrobial activity of LC-AMP-I1 *in vivo*

**Fig 9:**
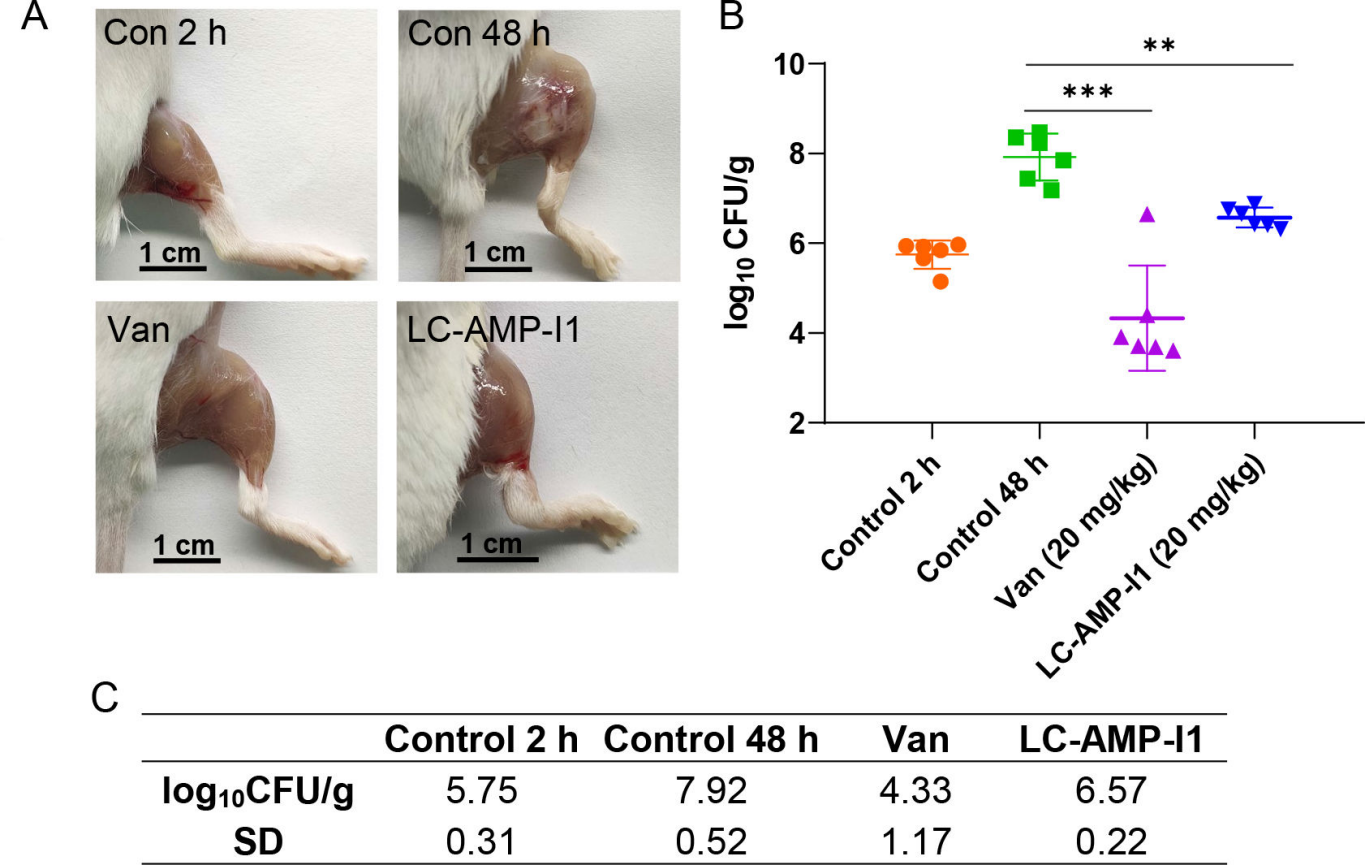
Antibacterial activity of LC-AMP-I1 *in vivo*. (**A**) Infection status of MRSA ATCC 43300 in mouse thigh. Mice in both the vancomycin-treated and LC-AMP-I1-treated groups were given intramuscular injection into the thigh at a dose of 20 mg/kg of body weight, and mice in the control 48-hour group were injected with phosphate-buffered saline (PBS). (**B**) Colony counts after the administration of PBS, vancomycin, or LC-AMP-I1 in neutropenic mouse thigh infection model. Each data point represents the value for a single mouse. All the experiments were performed in triplicate. Data are presented as mean values of 6 standard deviations. Data were statistically analyzed via ordinary one-way ANOVA; **P < 0.01; ***P < 0.001. (**C**) Each group’s average log_10_CFU/g and SD.

To evaluate the potency and safety of LC-AMP-I1, its *in vivo* antibacterial effect against MRSA was examined using a murine thigh infection model. As shown in Fig. 9A, compared with 2-hour post-infection, the thighs of mice in the control group exhibited notable swelling, erythema, and congestion at 48-hour post-infection, while the symptoms in the vancomycin group and the LC-AMP-I1 group were considerably diminished. The quantitative analysis indicated that LC-AMP-I1-treated mice exhibited a 1.35 log_10_CFU/g decrease in the average bacterial load of the thigh compared to the control group at 48 hours, while the vancomycin mice demonstrated a 3.59 log_10_CFU/g reduction (Fig. 9B). The results indicate that LC-AMP-I1 can effectively suppress bacteria proliferation *in vivo*. No death or notable toxic effects were observed in any experimental groups, confirming the *in vivo* biosafety of LC-AMP-I1.

### The antibacterial mechanism of LC-AMP-I1

**Fig 10:**
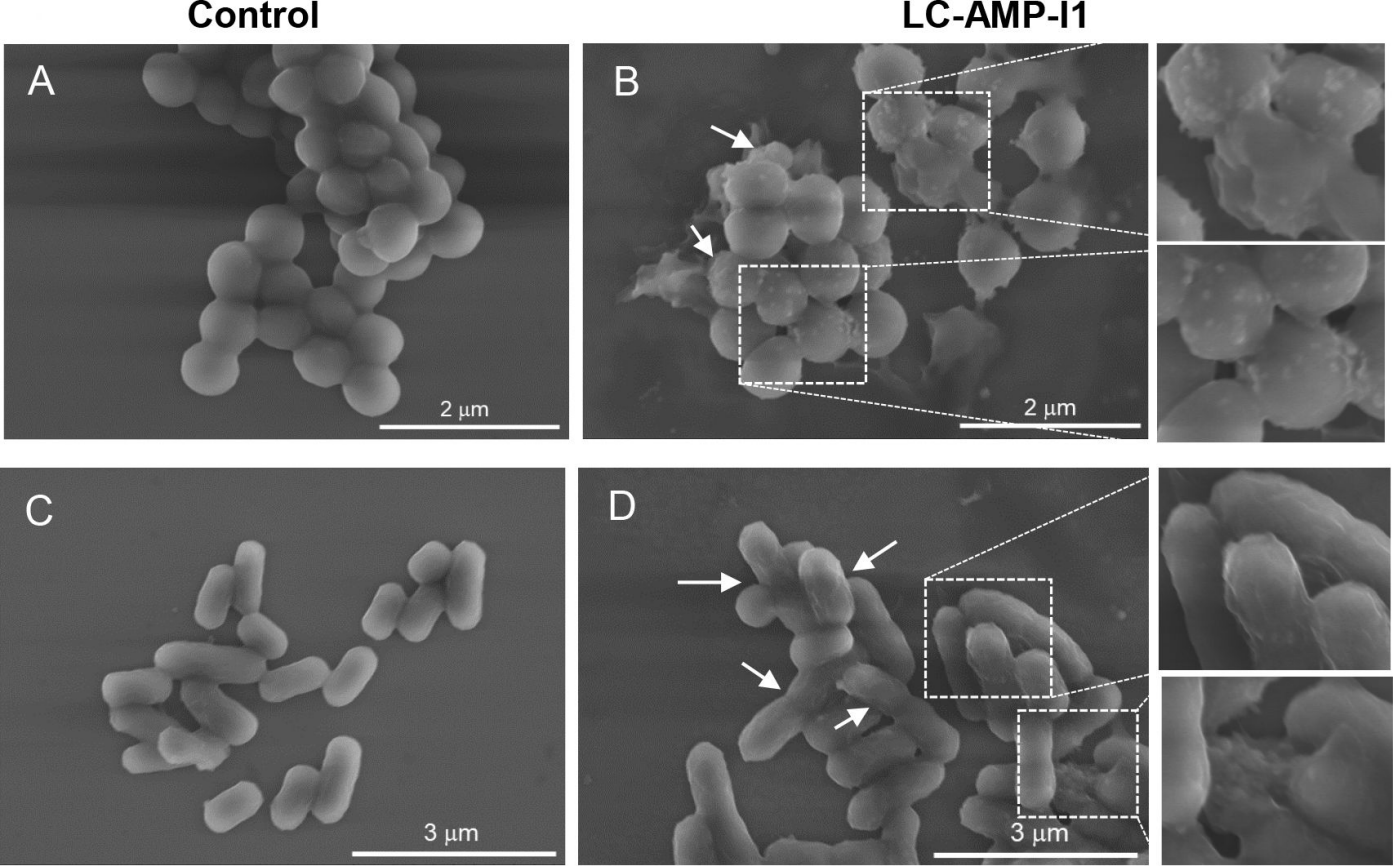
Scanning electron micrograph of *S. aureus* CMCC 26003 (**A and B**) and *E. coli* CCTCC AB 2018675 (**C and D**). The white arrows indicate the cell membrane damage.

The morphological structure of bacterial cell membranes was examined using scanning electron microscopy to investigate the potential mechanism of LC-AMP-I1 action on bacteria. As shown in Fig. 10, LC-AMP-I1 induced plasma membrane disruption in *S. aureus* after treatment for 30 min at 4× MIC, while the plasma membrane of untreated cells remained smooth and intact (Fig. 10A and B). Likewise, following treatment with LC-AMP-I1, the majority of *E. coli* cells displayed membrane rupture and pore formation (Fig. 10C and D).

**Fig 11:**
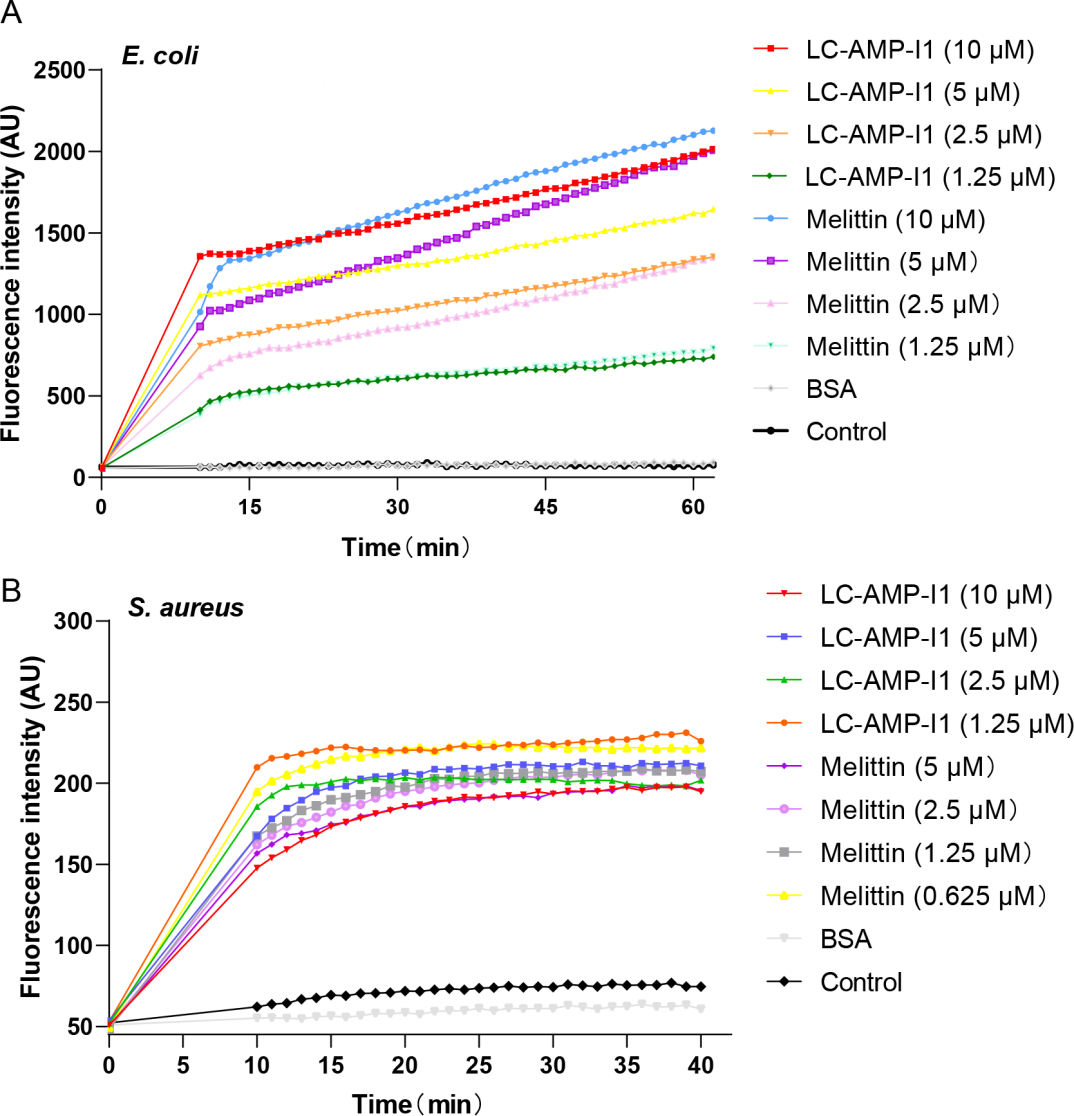
Effect of LC-AMP-I1 on bacterial cell membrane permeability. (**A**) *E. coli* CCTCC AB 2018675. (**B**) *S. aureus* CMCC 26003. Bacterial consumption of SYTOX Green was evaluated by detecting intracellular fluorescence every 1 min. AU, absorbance units. For the sake of clarity, error bars are not shown. One-way analysis of variance was used to analyze the data. Results show a statistically significant difference (*P* < 0.001).

To elucidate the impact of LC-AMP-I1 on bacterial cell membranes, we employed SYTOX Green, a nucleic acid dye incapable of permeating cell membranes, to assess its influence on the permeability of *E. coli* and *S. aureus* membranes. Consistent with the results observed with melittin, the fluorescence intensity of *E. coli* exhibited a time-dependent increase corresponding to the rising concentration of the peptide during the action of LC-AMP-I1. The findings indicated that LC-AMP-I1 could enhance the permeability of the *E. coli* cell membrane (Fig. 11A). However, the impact of LC-AMP-I1 on the cell membrane of *S. aureus* was more rapid (Fig. 11B). During peptide treatment, the intracellular fluorescence intensity stabilized after approximately 10 min. Based on the above morphological data, LC-AMP-I1 may act similarly to melittin, which directly affects bacterial cell membrane permeability at low concentrations and directly interferes and destroys structure-function at high concentrations.

## DISCUSSION

Venoms from diverse animals, such as snakes, bees, spiders, scorpions, and frogs, consist of various bioactive peptides (35, 36). Among 11 toxin-based molecules (including enzymes, peptides, and non-protein molecules) authorized for the pharmaceutical market, 7 are venom-derived peptides (37). In addition, there are over 15 venom-derived peptides undergoing clinical trials, including 4 AMPs (38). Melittin, the main component of *Apis mellifera* honeybee venom, has broad-spectrum antimicrobial activity and high hemolytic toxicity (39, 40). This amphiphilic α-helical peptide possesses anti-inflammatory properties in small doses by intradermal injection and is approved by the FDA for phase I/II clinical trials (41, 42). The findings of this study demonstrated that, in comparison to melittin, the venom-derived antimicrobial peptide LC-AMP-I1 exhibited equivalent or superior beneficial effects on the inhibitory activity, drug resistance, salt stability, biofilm inhibition and eradication against standard and clinical Gram-negative bacterial strains, as well as synergistic antibacterial activity at the same or higher molar concentrations (43, 44). Notably, it also displays low hemolytic activity and negligible cytotoxicity to human cells (45–48).

Wolf spiders (family *Lycosidae*) are globally distributed and occupy nearly all habitats. The venom of *Lycosidae* comprises numerous cationic antimicrobial peptides exhibiting various activities. The biological activities of 14 AMPs from *Lycosidae* venom are summarized in Table 2. Notably, our group identified the eight AMPs (Lycosin-I, Lycosin-II, LVTX-8, LVTX-9, LS-AMP-E1, LS-AMP-F1, XYP1, and LC-AMP-F1) and LC-AMP-I1 that were previously reported from the Chinese wolf spiders. These AMPs contributed nearly two-thirds of the studies in *Lycosidae* venom AMPs. All these *Lycosidae* AMPs possess α-helical structure and exhibit diverse inhibitory activities including antibacterial, antibiofilm, antifungal, anticancer, antiparasitic, and antiinflammatory effects. Meanwhile, most of these AMPs possess high toxicity toward mammalian cells (e.g., hemocytes). As shown in Table 2, only four peptides (LVTX-9, XYP1, LC-AMP-F1, and LC-AMP-I1) have negligible toxicity or non-toxicity for hemocytes. LVTX-9 derived from *Lycosa vittata* venom exhibited minimal inhibitory activity against malignant melanoma cells and negligible hemolytic activity at a concentration of 200 µM or 370.03 µg/mL (49). Thus far, we have successfully identified three AMPs from *L. coelestis* venom with low or no hemolytic activity. XYP1 exhibited potent anti-toxoplasma activity and 6.15% hemolysis at 160 µM or 463.96 µg/mL (25), and LC-AMP-F1 with antibacterial and antibiofilm activities showed nonhemolytic activity in 160 µM or 310.79 µg/mL (26). In this work, the third venom-derived AMP LC-AMP-I1 from this wolf spider inhibited bacterial growth and biofilm formation, exhibiting only 10.13% hemolysis at 320 µM or 987.75 µg/mL and mild toxicity to normal human cells. It is essential to note that AMPs exhibiting broad-spectrum antibacterial activity primarily target cell membranes. Despite the disparities in cell membranes between bacteria and humans, both possess phospholipids as their primary component. It is necessary to identify template molecules for prospective new antimicrobial peptide drugs that target bacterial cells while exhibiting low toxicity to human cells. Consequently, the low toxicity and antibacterial properties of LC-AMP-I1 will enable it to serve as a valuable lead compound for the development of antimicrobial peptide drugs.

**TABLE 2:** Biological activities of *Lycosidae* venom AMPs^*a*^

Name	Source	Amino acid sequence	Helical content (%)	Activity	Hemolysis (%) (concentration)	References
Lycocitin 1	*L. singoriensis*	GKLQAFLAKMKEIAAQTL-NH_2_	100.00	B, F	n.d.	(50)
Lycocitin 2	*L. singoriensis*	GRLQAFLAKMKEIAAQTL-NH_2_	100.00	B, F	n.d.	(50)
Lycosin-I^*b*^	*L. singoriensis*	RKGWFKAMKSIAKFIAKEKLKEHL-NH_2_	91.67	B, F, Bf, C, P, I	37.00 (200 µM or 577.34 µg/mL)	(19–21, 51–53)
Lycosin-II^*b*^	*L. singoriensis*	VWLSALKFIGKHLAKHQLSKL-NH_2_	95.24	B, F, Bf, I	20.00 (50 µM or 120.82 µg/mL)	(54–56)
LyeTx I	*Lycosa erythrognatha*	IWLTALKFLGKNLGKHLAKQQLAKL-NH_2_	100.00	B, F	50.00 (130 µM or 368.12 µg/mL)	(57)
LVTX-8^*b*^	*L. vittata*	IWLTALKFLGKNLGKHLAKQQLSKL-NH_2_	100.00	C	66.70 (5 μM or 14.24 µg/mL)	(58, 59)
LVTX-9^*b*^	*L. vittata*	ASIGALIQKAIALIKAKAA-NH_2_	100.00	C	0.00 (200 µM or 370.03 µg/mL)	(49)
LS-AMP-E1^*b*^	*L. sinensis*	AGMKNIIDAIKKKLGGKL-NH_2_	72.22	B, Bf	34.92 (200 µM or 379.43 µg/mL)	(23)
LS-AMP-F1^*b*^	*L. sinensis*	TGLGKIGYLMKKLLSKAKV-NH_2_	89.47	B, Bf	17.00 (200 µM or 409.45 µg/mL)	(23)
XYP1^*b*^	*L. coelestis*	KIKWFKAMKSIAKFIAKDQLKKHL-NH_2_	95.83	P	6.15 (160 µM or 463.96 µg/mL)	(25)
LC-AMP-F1^*b*^	*L. coelestis*	AGLGKIGALIQKVIAKYKA-NH_2_	78.95	B, Bf	0.00 (160 µM or 310.79 µg/mL)	(26)
LC-AMP-I1^*b*^	*L. coelestis*	GRMQEFIKKLKAYLRKMKEKFSQIS-NH_2_	96.00	B, Bf, C	10.13 (320 µM or 987.75 µg/mL)	This work
Lycotoxin I	*Hogna carolinensis*	IWLTALKFLGKHAAKHLAKQQLSKL-NH_2_	100.00	B, F	55.00 (200 µM or 568.54 µg/mL)	(60)
Lycotoxin II	*Hogna carolinensis*	KIKWFKTMKSIAKFIAKEQMKKHLGGE-OH	77.78	B, F	n.d.	(60)

^*a*^ Biological activities of *Lycosidae* venom AMPs are indicated as follows: B, antibacterial; Bf, antibiofilm; F, antifungal; C, anticancer; P, antiparasitic; I, antiinflammatory; and n.d., no data available.

^*b*^ Peptide that was identified in our previous or this study by our group.

Nevertheless, akin to the majority of issues encountered in the advancement of novel antimicrobial peptides, the investigation and utilization of LC-AMP-I confront three primary challenges. First, the *in vivo* activity validation still requires more experimental data from different animal models, including systematic comparisons of different routes of administration. Second, although we found that LC-AMP-I1 disrupted bacterial biofilm, we are unable to ascertain whether its mechanism of action involves direct targeting of the cell membrane or a stage in the cascade that follows. Therefore, comprehensive mechanistic investigations are imperative in future research. Finally, the production cost of LC-AMP-I1 must be further reduced for better and faster application. Sequence deletion, modification, optimization of chemical synthesis, and construction of biosynthetic systems are crucial for the market transformation of LC-AMP-I1.

## MATERIALS AND METHODS

### Peptide purification, MALDI-TOF/TOF analyses, and cDNA library construction of *L. coelestis* venom gland

The wolf spider *L. coelestis* was captured in Jiangxi Province, China. Venom sample (0.5 mg) preparation, RP-HPLC separation, analyses of MALDI-TOF/TOF mass spectrometry, MS/MS for the eluted fractions, venom gland cDNA library construction, and DNA sequencing were conducted following the protocol previously used by our group (22).

### Analyses of sequence, structure, and physical and chemical parameters

cDNA sequences were translated into amino acid sequences by the MEGA 7.0 software, and signal peptides of peptide precursors were predicted with the SignalP-6.0 Server (http://www.cbs.dtu.dk/services/SignalP/). Propeptide cleavage sites were confirmed by mature peptide sequences from MALDI-TOF/TOF sequencing, and mature peptide sequences were searched in the antimicrobial peptide database (https://aps.unmc.edu/) for homology prediction of AMPs. The secondary structure of LC-AMP-I1 was predicted by I-TASSER (https://zhanggroup.org/I-TASSER/), and physiochemical characteristics of the peptides were assessed using ProtParam (https://web.expasy.org/protparam/), while the helical wheel projection of the peptide was conducted using HeliQuest (https://heliquest.ipmc.cnrs.fr/).

### Peptide synthesis

LC-AMP-I1 was obtained from CHENPEPTIDE (Nanjing, China) and synthesized in solid phase using standard 9-fluoromethoxy carbonyl chemistry.

### Materials and strains of bacteria

*Salmonella typhimurium* CGMCC 1.1174, *Shigella dysenteriae* CGMCC 1.1869, and *Pseudomonas aeruginosa* CGMCC 1.596 were obtained from the China General Microbiological Culture Collection Center. *Staphylococcus aureus* CMCC 26003 was acquired from the National Center for Medical Culture Collections. Methicillin-resistant *Staphylococcus aureus* ATCC 43300 was obtained from the American Type Culture Collection.

All clinical isolates examined in this investigation were obtained between October 2021 and April 2023 from the Affiliated Nanhua Hospital of the University of South China. Multidrug-resistant strains of *Enterococcus faecium*, *S. aureus*, *Klebsiella pneumoniae*, *Acinetobacter baumannii*, *P. aeruginosa*, and *Enterobacter* species were isolated from patients’ secretions, urine, sputum, or blood. LO2, HepG2, and HONE-1 cells were obtained from the Cancer Research Institute of the University of South China. The 4T1 and the HEK293T cells were bought from the American Type Culture Collection (ATCC).

Twenty-four KM mice (5–6 weeks) were acquired from Hunan SJA Laboratory Animal Co., Ltd (Changsha, China). Cell culture media Dulbecco’s modified Eagle’s medium, various bioactive peptides I 1640, penicillin–streptomycin, and fetal bovine serum (FBS) were purchased from Gibco (Carlsbad, USA). Erythromycin, levofloxacin, and trifluoroethanol (TFE) were purchased from Aladdin Industrial Inc. (Shanghai, China).

### Circular dichroism analysis

The secondary structures of LC-AMP-I1 were investigated by CD (JASCO J-1500 spectropolarimeter). The peptide was dissolved in water and a 50% (vol/vol) solution of TFE, and spectra were recorded from 260 to 190 nm. The peptide concentration was 500 µg/mL, whereas the quartz test tube had an optical diameter of 10 mm. Three measurements were conducted for each sample, and the resultant CD spectra were then transformed into mean residue ellipticity using the provided equation:


$$\theta_{M}=(\theta_{obs}\times \;1,000)/(c\;\times \;l\;\times \;n).$$

Based on the equation, *θ*_obs_ is the experimentally measured ellipticity, *c* is the peptide concentration in mM, *l* is the path length of the cell in millimeters, and *n* is the number of amino acids.

### Minimum inhibitory concentration assays

The MIC values of peptides were determined using the broth microdilution method with reference to the Clinical and Laboratory Standards Institute protocol. Standard strains and clinical isolates of drug-resistant strains were grown in Mueller-Hinton broth until they reached the exponential growth phase. The bacterial solution underwent a dilution with MH broth to produce a total colony count of about 10^5^ CFU/mL. In 96-well plates (Corning, NY, USA), LC-AMP-I1 was diluted twofold with bacterial suspension at concentrations ranging from 1.25 to 40 µM and then the peptide solution was incubated for 16–18 hours at 37°C. The positive controls were subjected to incubation with melittin, whereas the negative controls were treated with phosphate-buffered saline (PBS).

OD_600_ was measured using a Cytation3 multifunctional microplate detector (Bio-Tek, Vermon, USA) and the inhibition rate was calculated by the following formula:


$$\%\;inhibition=(A_{control}-A_{sample})/A_{control}.$$

### Drug resistance assay

The detection of bacterial resistance was accomplished by evaluating the MIC values of *E. coli* CCTCC AB 2018675 and *S. aureus* CMCC 26003 after a continuous 21-day exposure to the peptide. The MIC values of LC-AMP-I1 against *E. coli* CCTCC AB 2018675 and *S. aureus* CMCC 26003 were first established. Next, the bacteria in a peptide solution with 1/2× MIC were introduced into MH broth and placed in a constant temperature shaker at 37°C for incubation. On the following day, the bacteria were allowed to proliferate until they reached the logarithmic phase, followed by a subsequent MIC evaluation, which was carried out continuously for a period of 21 days. Chloramphenicol and ampicillin were used as positive controls.

### Time–kill kinetics

*E. coli* CCTCC AB 2018675 and *S. aureus* CMCC 26003 were cultured to 10^7^ CFU/mL in MH broth. The bacterial suspension was then incubated with LC-AMP-I1 solution (final concentrations of 1×, 4×, and 8× MIC). The bacterial suspensions were diluted and spread on LB agar at 0, 15, 30, 60, 90, and 120 min. After incubation at 37°C for 24 hours, the number of colonies was counted as log_10_ CFU/mL. Each treatment was repeated three times, and melittin was used as a positive control.

### Biofilm inhibition and eradication activity

*E. coli* CCTCC AB 2018675 and *S. aureus* CMCC 26003 were cultured at 37°C and 200 r/min overnight, and when the bacteria grew to the logarithmic growth phase, they were diluted to about 10^5^ CFU/mL with RPMI-1640 medium containing 10% FBS.

The biofilm inhibition assays were conducted as follows. LC-AMP-I1 or melittin solution (final concentration of 0.25–4× MIC) was serially diluted twofold and then added into 96-well plates containing bacterial solutions, with a final volume of 200 µL. The plates were then incubated for a duration of 24 hours at 37°C and washed three times with PBS to remove planktonic bacteria. After drying at room temperature, methanol was added and fixed for 15 min, and then the methanol was aspirated. Each well received 200 µL of 0.1% (g/v) crystal violet (BBI, Shanghai, China) solution, followed by staining for 10 min and washing three times with sterile water. Finally, 200 µL of 33% (vol/vol) acetic acid was added to each well to dissolve the crystal violet, and the absorbance at 595 nm was measured.

The biofilm eradication assays were conducted as follows. The bacterial solution was incubated for 24 hours in 96-well plates. After three washes with PBS to eliminate planktonic bacteria, 0.25–4× MIC of LC-AMP-I1 or melittin was added to RPMI-1640 medium containing 10% FBS (final concentration of 0.25–4× MIC) and incubated for 24 hours in 96-well plates. The same methods were applied to the biofilm as previously mentioned.

### Stability analysis

In order to assess the impact of salt ions on the efficacy of LC-AMP-I1 against *E. coli* CCTCC AB 2018675 and *S. aureus* CMCC 26003, the MIC values were measured in the presence of physiological amounts of various salts (150 mM NaCl, 4.5 mM KCl, 6 µM NH_4_Cl, 1 mM MgCl_2_, or 2.5 mM CaCl_2_) using the aforementioned methodology.

To assess the stability of LC-AMP-I1 with respect to temperature and pH, the peptides were subjected to treatment in a water bath at 80°C or 100°C for 1 hour, and the pH of the medium was varied to values from 5 to 9. The MIC values were then obtained, and each measurement was replicated three times.

### Hemolytic assay

The erythrocytes of rabbits were washed three times with PBS and then resuspended to obtain a 1% erythrocyte suspension in PBS. The solution containing LC-AMP-I1 or melittin at a final concentration ranging from 5 to 320 µM was introduced into the prepared suspension of erythrocytes. After incubation at 37°C for 1 hour, this solution was centrifuged at 930 *× g* for 5 min, and the supernatant was transferred to a new 96-well plate. By measuring absorbance at 570 nm, hemoglobin release was monitored. 100% hemolysis was measured using 0.1% (vol/vol) Triton X-100 (Sigma-Aldrich, MO, USA), and 0% hemolysis was measured using PBS.

### Cytotoxicity assay

The cytotoxicity of LC-AMP-I1 or melittin against 4T1, HepG2, HONE-1, LO2, and HEK293T cell lines was assessed using the CCK-8 test (BBI, Shanghai, China). Briefly, 10^4^ cells per well were inoculated in 96-well plates, and after the cells adhered to the wall, various final concentrations of peptides (0.625–20 μΜ) were added and incubated for 12 hours at 37°C under a 5% CO_2_ atmosphere. The absorbance at 450 nm was determined to calculate the cell viability.

### Synergistic activity assay

Combined chemosensitivity test was determined by the checkerboard method using six dilutions, with the maximal concentration of 2× MIC for peptides and antibiotics, respectively. *E. coli* CCTCC AB 2018675 and *S. aureus* CMCC 26003 were used as test strains.

In brief, twofold serial dilutions with varied concentrations of antibiotics and peptides were prepared in a 96-well plate. After 16–24 hours of incubation at 37°C, the OD_600_ of bacterial suspensions was measured. The fractional inhibitory concentration index was computed using the following formula:

FICI = MIC of peptides in combination/MIC of peptides alone + MIC of antibiotics in combination/MIC of antibiotics alone.

The calculated FICI was interpreted as synergistic (FICI ≤ 0.5), additive (0.5 < FICI < 1), indifferent (1 ≤ FICI < 4.0), and antagonistic (FICI ≥ 4.0).

### Neutropenic mouse thigh infection model

A neutropenic mouse thigh infection model was established using female KM mice. Briefly, mice (*n* = 6 per group) received intraperitoneal injections of cyclophosphamide (150 and 100 mg/kg of body weight on days 4 and 1, respectively) prior to bacterial infection, resulting in neutropenia.

A bacterial suspension of MRSA ATCC 43300 was prepared by dilution with fresh MH broth. Each mouse was infected by intramuscular injection of 50 µL (1.5 × 10^6^ CFU) of bacterial fluid into the right thigh. Following a 2-hour period of infection, mice were given an intramuscular injection of either 50 µL of LC-AMP-I1 at a concentration of 20 mg/kg of body weight or an equivalent dose of vancomycin. The control group received an injection of an equivalent amount of PBS. Baseline bacterial load was established by executing untreated control animals at the beginning of the therapy. The mice were sacrificed at 48-hour post-infection, and the right thigh was aseptically excised. It was then submerged in sterile saline and homogenized using a homogenizer. The tissue sample was appropriately diluted in sterile saline solution and thereafter plated onto LB agar plates. The plates were then incubated at 37°C for 24 hours to facilitate the enumeration of bacterial colonies.

### Scanning electron microscopy

*E. coli* CCTCC AB 2018675 and *S. aureus* CMCC 26003 were grown to the exponential phase and incubated for 30 min with LC-AMP-I1. After 10 min of centrifugation at 1,000 *× g*, the cells were washed three times with saline and fixed overnight at 4°C with 4% paraformaldehyde. After three additional treatments with saline, the bacteria were dehydrated at a series of ethanol concentrations ranging from 20% to 100% and allowed to stand for 10 min between each application. Subsequently, the samples were immersed in 100% ethanol. The specimens were attached to aluminum ends. After depositing gold by sputtering, they were examined by a S-4800 scanning electron microscope (Hitachi, Tokyo, Japan).

### Cell membrane permeability assay

Peptide-induced damage to bacterial cell membranes was detected by SYTOX Green staining (61). *E. coli* CCTCC AB 2018675 and *S. aureus* CMCC 26003 in the logarithmic growth phase were rinsed three times with saline and then suspended in saline. The bacterial solution concentration was adjusted to 5 × 10^7^ CFU/mL. The bacterial suspension was mixed with 5 µM SYTOX Green (KeyGEN BioTECH, Jiangsu, China) and incubated for 15 min at room temperature in the dark. The mixture was added to a black 96-well plate at a density of 100 µL/well, and subsequently, peptide solution (final concentration of 1×, 2×, or 4× MIC) was added to the wells. Melittin was used as the positive control, and bovine serum albumin served as the negative control. The uptake of SYTOX Green was monitored using a Cytation3 multifunctional microplate detector (Bio-Tek, Vermont, USA) with excitation and emission wavelengths of 485 and 520 nm, respectively.

## SUPPLEMENTAL MATERIAL

Supplemental material aac.00424-24-s0001.docx Figures S1 to S5; Table S1.

ASM does not own the copyrights to Supplemental Material that may be linked to, or accessed through, an article. The authors have granted ASM a non-exclusive, world-wide license to publish the Supplemental Material files. Please contact the corresponding author directly for reuse.
