# Supplementary material for: LC-AMP-I1, a novel venom-derived antimicrobial peptide from the wolf spider Lycosa coelestis
Source: Antimicrob Agents Chemother. 2024 Dec 2;69(1):e00424-24. doi: 10.1128/aac.00424-24 (PMC11784185; doi:10.1128/aac.00424-24)
Supplement: Supplemental material — Figures S1 to S5; Table S1. [file aac.00424-24-s0001.docx]

**Supplementary Material**

**LC-AMP-I1, a novel venom-derived antimicrobial peptide from the wolf spider *Lycosa coelestis***

Junyao Wang ^a, 1^, Xi Liu ^a, 1^, Yuxin Song ^a^, Zhonghua Liu ^c^, Xing Tang ^b, *^ and Huaxin Tan ^a, *^

^a^Institute of Biochemistry and Molecular Biology, Hengyang Medical College, University of South China, Hengyang 421001, China;

^b^Hunan Key Laboratory for Conservation and Utilization of Biological Resources in the Nanyue Mountainous Region, College of Life Sciences, Hengyang Normal University, Hengyang 421002, China;

^c^The National and Local Joint Engineering Laboratory of Animal Peptide Drug Development, College of Life Sciences, Hunan Normal University, Changsha 410081, China.

^1^These authors contributed equally to this work.

*Corresponding authors. xtang2011@sina.com (X.T.); huaxintan@usc.edu.cn (H.T.)

E-mail addresses: [20212013110983@stu.usc.edu.cn](mailto:20212013110983@stu.usc.edu.cn) (J. W.), [20222013111190@stu.usc.edu.cn](mailto:20222013111190@stu.usc.edu.cn) (Y.S.), [20232023111520@stu.usc.edu.cn](mailto:20232023111520@stu.usc.edu.cn) (X.L.), [liuzh@hunnu.edu.cn](mailto:liuzh@hunnu.edu.cn) (Z.L.).

**
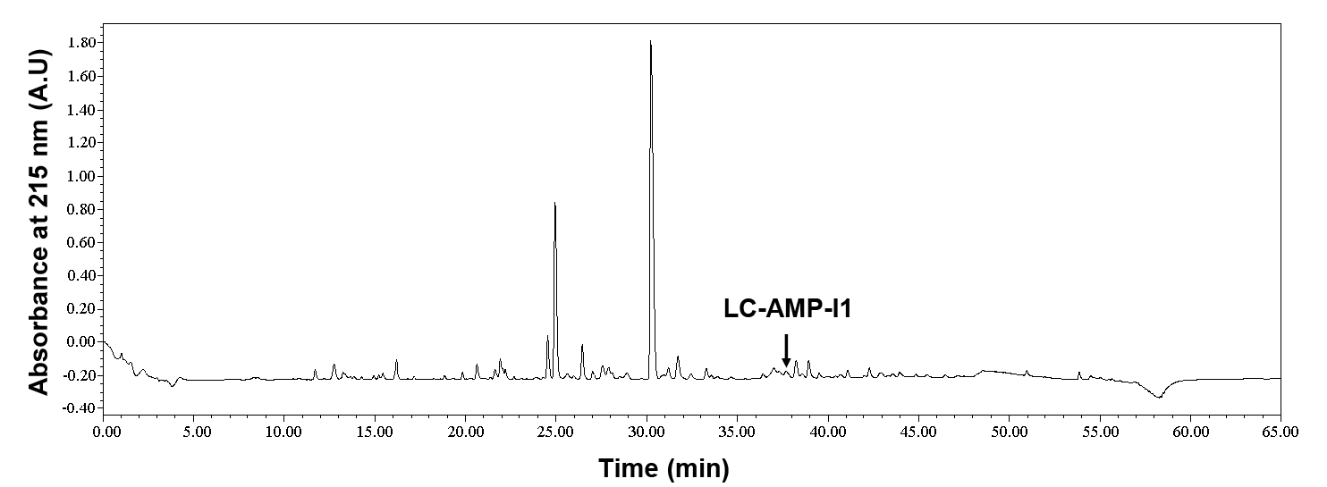
**

Figure S1. Complete RP-HPLC profiles of *L. coelestis* venom peptides.





Figure S2. LC-AMP precursor 3 of antimicrobial peptides (LC-AMP-I1 and LC-AMP-D family) from *L. coelestis* venom glands. The signal peptide was in italic and the propeptides were underlined. The mature peptides were bold and the peptide names were above the mature sequences. Rectangles denoted the additional residues (GR) in C-terminal of mature peptides.

Table S1. The clinical profiles of MDR strains isolated from 18 cases.

| **Species** | **Sample number** | **Resistance type** | **Age  range** | **Disease** | **Specimen  type** |
| --- | --- | --- | --- | --- | --- |
| ***E. faecium*** | 20220505117 | None | 80-90 | UTI | urine |
|  | 20220105146 | None | 20-30 | acute pancreatitis | blood |
|  | 20220110132 | None | 30-40 | UTI | urine |
| ***S. aureus*** | 20211020125 | MRSA | 50-60 | diabetic foot | excretion |
|  | 20211011106 | MRSA | 30-40 | pregnancy | excretion |
|  | 20210930047 | MRSA | 60-70 | injury | sputum |
| ***K. pneumoniae*** | 20211017048 | CR | 40-50 | open injuries | excretion |
|  | 20230418088 | CR | 30-40 | uremia | excretion |
|  | 20230419026 | ESBLs | 50-60 | Multiple injuries | sputum |
| ***A. baumannii*** | 20211018150 | CR | 50-60 | buring | excretion |
|  | 20211005014 | CR | 60-70 | stroke | sputum |
|  | 20211012080 | CR | 70-80 | abdominal pain | blood |
| ***P. aeruginosa*** | 20211104085 | CR | 80-90 | pneumonia | sputum |
|  | 20211115011 | CR | 50-60 | aneurysm | sputum |
|  | 20211031041 | CR | 80-90 | pneumonia | sputum |
| ***E. coli*** | 20211028005 | ESBLs | 70-80 | abdominal pain | blood |
|  | 20220223151 | CR | 70-80 | prostatic hyperplasia | urine |
|  | 20211019073 | ESBLs | 70-80 | notalgia | urine |

MRSA: Methicillin-resistant *Staphylococcus aureus*; CR: Carbapenem-Resistant; ESBLs: extended spectrum beta-lactamases; UTI: urinary tract infection.


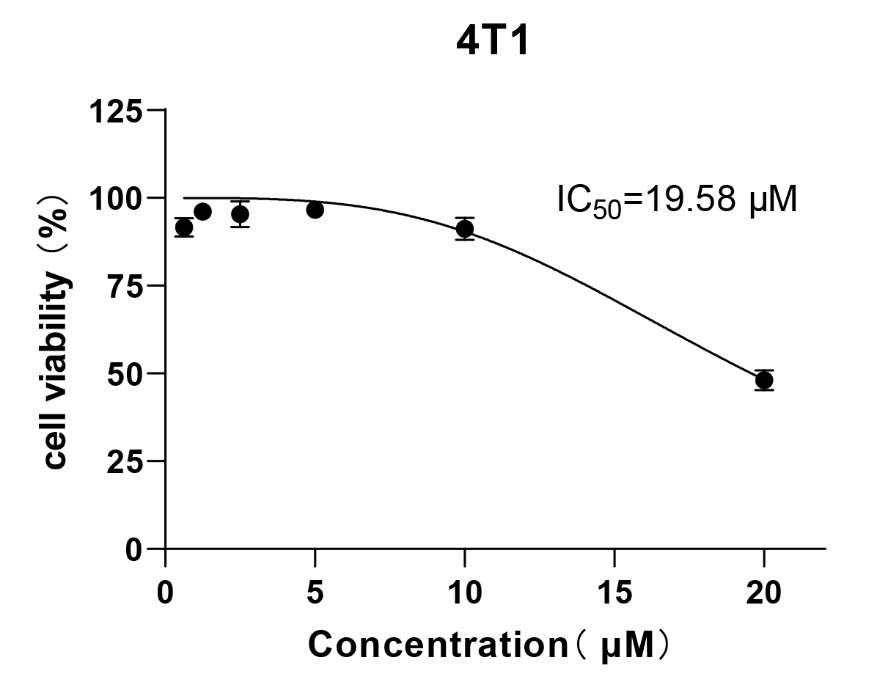


Figure S3. IC_50_ value for LC-AMP-I1 versus 4T1 cells.


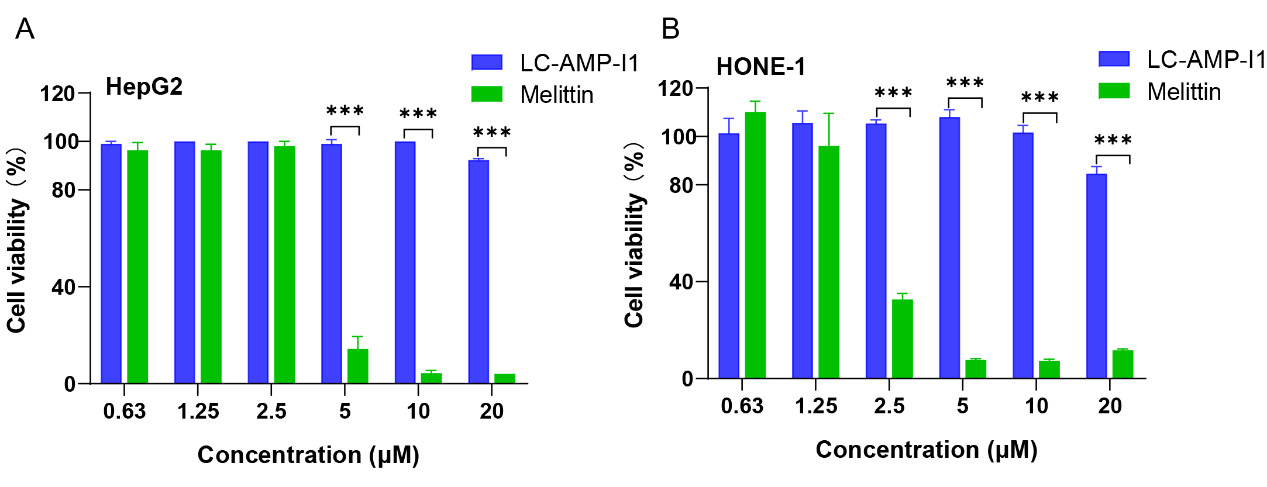


Figure S4. Cytotoxicity of LC-AMP-I1 to HepG2 cells (A) and HONE-1 cells (B).


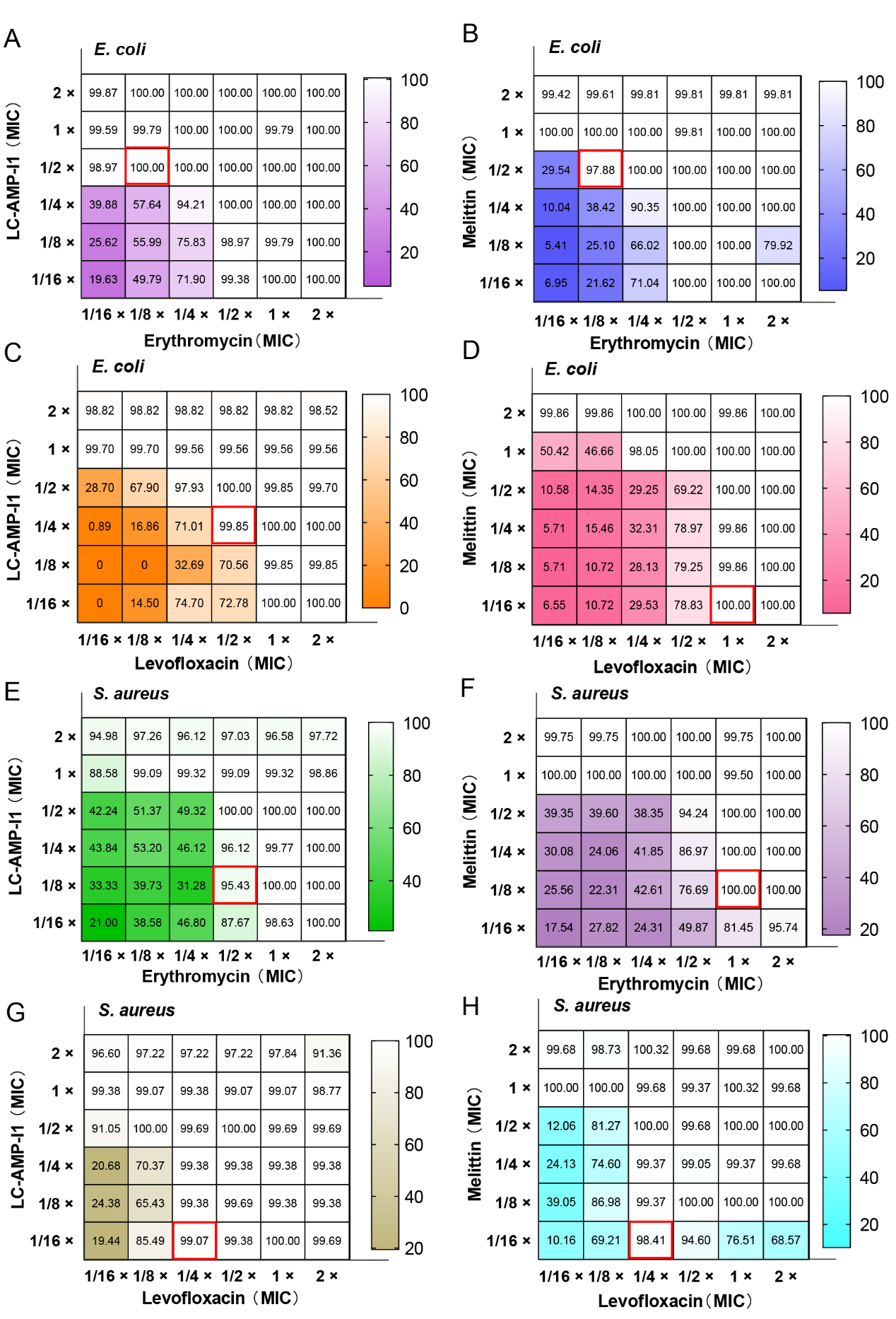
Figure S5. Checkerboard experiment in *E. coli* and *S. aureus*. The concentrations of LC-AMP-I1 and melittin in combination with erythromycin (A, B, E, and F) and levofloxacin (C, D, G, H) were determined, respectively.
